# Risk factors for loss of Varicella immunity after pediatric kidney transplantation

**DOI:** 10.1007/s00467-025-07022-7

**Published:** 2025-11-13

**Authors:** Helen Pizzo, Priya R. Soni, Santhosh Nadipuram, James Mirocha, Jonathan Garrison, Sherlyn Hilario, Dechu Puliyanda

**Affiliations:** 1 Cedars-Sinai Medical Center Guerin Children’s, 8700 Beverly Blvd., Los Angeles, CA 90048 USA; 2 Pediatric Infectious Disease, Cedars-Sinai Medical Center, Los Angeles, USA; 3 Biostatistics Core, Research Institute, Cedars-Sinai Medical Center, Los Angeles, USA

**Keywords:** Infection, Pediatric, Seroconversion, Transplant, Varicella zoster, Immunosuppression

## Abstract

**Background:**

Varicella zoster (VZV) vaccination pre-kidney transplant (Tx) can help prevent severe disseminated VZV in immunosuppressed recipients; however, studies have shown loss of humoral immunity post-Tx.

**Methods:**

A retrospective analysis of 45 pediatric kidney Tx recipients with positive pre-Tx VZV IgG (>1.09 index). VZV IgG was assessed annually and compared with the induction agent used, the number of VZV vaccines received, and the interval between the last dose of VZV vaccine and Tx.

**Results:**

Median age at Tx was 16.7 years (IQR 12.7–18.5). 11 of 45 (24.4%) patients lost immunity to VZV at a median of 12.6 months post-Tx. Those who lost VZV immunity were younger at the time of Tx, 12.4 years vs. 17.3 years (*P* = 0.05) and more likely to be on steroid-based immunosuppression 81.8% vs. 32.4% (*P* = 0.006). There were no differences between the induction agents used and the ability to maintain VZV IgG antibodies. Subjects who required ≥3 doses of VZV vaccine to develop VZV IgG seropositivity were at a higher risk for losing their anti-varicella antibody post-Tx (HR 3.81, 95% CI 1.09–13.30, *P* = 0.04). Receiving VZV vaccination <1 year prior to kidney Tx was associated with a higher risk for losing anti-varicella antibody after Tx (HR 6.97, 95% CI 2.08–23.34).

**Conclusion:**

In this small cohort, pediatric kidney Tx recipients are more likely to lose VZV IgG in those who were younger at the time of Tx, on steroid-based immunosuppression, required 3 or more doses of VZV vaccination to seroconvert, or received VZV vaccine <1 year before Tx.

**Graphical abstract:**

**Figure:**
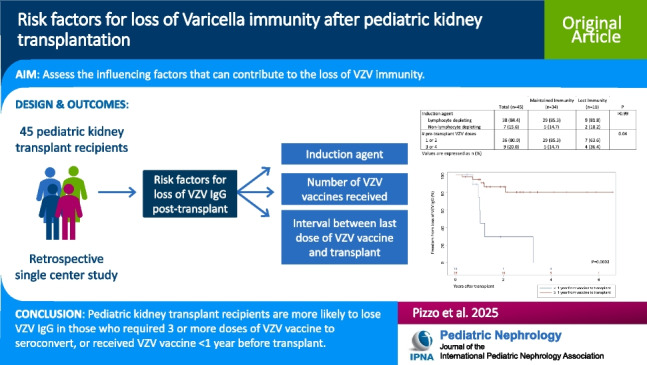
A higher resolution version of the Graphical abstract is available as Supplementary information

**Supplementary Information:**

The online version contains supplementary material available at 10.1007/s00467-025-07022-7.

## Introduction

Guidelines for vaccination of solid organ transplant candidates advise that every effort should be made to ensure completion of recommended pre-transplant vaccines prior to surgery, as immunosuppressed transplant recipients are at higher risk for invasive disease and are less likely to produce as robust an immune response to vaccines compared to those who are not immunocompromised [1–3]. With gaps in herd immunity secondary to a lack of or incomplete vaccination in the community, the risk for exposure to vaccine-preventable pathogens increases [4, 5]. In a multi-center retrospective study of pre-kidney transplant vaccination coverage among 254 European children, unvaccinated children were four times more likely to acquire a vaccine-preventable illness post-transplant compared to those who were vaccinated [6]. Post-transplant vaccine-preventable infections have also been shown to be associated with morbidity, mortality, and higher costs. A retrospective study of 6980 children across 45 pediatric transplant centers in the United States found more than 15% of solid organ transplant recipients were hospitalized with a vaccine-preventable illness in the first 5 years after transplant, with 1.7% mortality, and costing $120,498 more than transplant-related hospitalizations not associated with vaccine-preventable infections [7]. Furthermore, the authors of this study noted that 2.1% of hospitalizations in the first 5 years after pediatric solid organ transplant were due to varicella zoster (VZV) infections — a rate similar to the cohort admitted for illnesses due to pneumococcus (2.0%) and respiratory syncytial virus (1.8%) [7].

The efficacy of the recommended 2-dose varicella vaccine among the general pediatric population ranges between 94 and 98% [8, 9]. Vaccination against VZV and monitoring for antibody response among kidney transplant candidates can help prevent complications associated with varicella infection, which in immunosuppressed transplant recipients can include cutaneous, visceral, and systemic disease [10, 11]. In a study of 704 children who received a kidney transplant, varicella disease was only observed in those who did not develop or lost their VZV antibodies [12]. Despite confirmation of serologic response to vaccinations pre-transplant, studies have shown loss of humoral immunity post-transplant. In a retrospective single-center study of 18 pediatric solid organ transplant recipients with confirmed VZV seropositivity prior to transplant, 11.1% of the cohort lost their anti-varicella antibody within 6 months following transplant [13]. Broyer et al. noted that among 97 children who received VZV vaccination prior to kidney transplantation, 7% had negative VZV immune globulin G (IgG) at 1 year; this number increased to 24% at 5 years post-transplant [12]. While some studies in adult kidney transplant recipients found 100% VZV IgG positivity up to 1080 days after transplant, others mirror the findings in pediatric research with declining rates of VZV IgG seropositivity [14–18]. Potential influences for losing VZV immunity include the age at the time of transplant, the number of VZV vaccines received prior to transplantation, and the time interval between VZV vaccination and transplant [19–22]. In this study, we assessed the potential risk factors that could contribute to the maintenance of or loss of VZV immunity after pediatric kidney transplantation: (1) induction agent used, (2) number of VZV vaccines received prior to transplant, (3) interval between the last dose of VZV vaccine and transplantation.

## Methods

### Patient population

This is a single center retrospective observational study of 45 pediatric patients aged 2–20 years who received a kidney transplant between April 2018 and September 2023. All recipients who received pre-transplant varicella vaccination with documentation of subsequent positive VZV IgG (>1.09 index) and received post-transplant VZV IgG monitoring were included in the study. Patients received induction immunosuppression with a lymphocyte-depleting agent (LDA; anti-thymocyte globulin (ATG) or alemtuzumab) or a non-lymphocyte-depleting agent (NLDA; basiliximab) and were maintained on mycophenolate mofetil, tacrolimus, with or without steroids. No subjects had a history of natural VZV infection before or after kidney transplant. Median follow-up duration was 14.9 months post-transplant (IQR 11.9–32.0 months).

### VZV IgG testing

Pre-transplant VZV IgG, determined by enzyme immunoassay, was assessed at the initial pre-transplant evaluation and reassessed every 2 years until transplant. Those who were seronegative at any point prior to transplant received an additional VZV vaccine, and VZV serology was repeated at least 4 weeks following vaccination; a maximum of 4 doses of VZV vaccine was given per patient. Post-transplant VZV IgG monitoring was performed every 6–12 months post-transplant. VZV IgG was considered positive when the value was >1.09 index. Equivocal titers (0.91–1.09) were considered negative in our analysis.

### Statistical analysis

Numerical variables were summarized by median and interquartile range (IQR) and were compared across groups by the Wilcoxon rank sum test. Categorical variables were summarized by frequency and percentage and were compared across groups by the Fisher exact test. Freedom from loss of VZV IgG was estimated by the Kaplan–Meier method, and group differences were assessed by the log-rank test. Non-adjusted hazard ratios (HR) and their 95% confidence intervals (CI) were obtained from Cox proportional hazards models. A two-sided 0.05 significance level was used throughout. SAS version 9.4 (SAS Institute, Cary, North Carolina) was used for statistical calculations.

This study was approved by the Cedars-Sinai Medical Center Institutional Review Board (STUDY00000398). This study was conducted in accordance with ethical guidelines based on federal regulations and the common rule. Cedars-Sinai Medical Center also has a Federal Wide Assurance.

## Results

A total of 45 subjects received pre-transplant varicella vaccination with confirmation of seroconversion prior to kidney transplantation and received post-transplant VZV IgG monitoring starting at 6–12 months following surgery — this represented 100% of pediatric renal transplants performed at our center during the study’s inclusion period. Median age at transplant was 16.7 years (IQR 12.7–18.5), 64.4% were males, 64.4% were Hispanic, 88.9% received a deceased-donor renal transplant, and 13.3% were highly sensitized (PRA > 30%). Comparison of patient characteristics among those who maintained post-transplant VZV immunity (*n* = 34) vs. those who lost immunity (*n* = 11) is highlighted in Table 1. Notably, those who lost VZV immunity were younger at the time of transplant, 12.4 years (IQR 3.9–17.4) vs. 17.3 years (IQR 13.4–19.0) (*P* = 0.05), and more likely to be on steroid-based immunosuppression, 9 (81.8%) vs. 11 (32.4%) (Table 1, *P* = 0.006). Furthermore, those who were highly sensitized were not at higher risk for losing their anti-varicella antibody following transplant (Table 1, *P* = 0.15).

**Table 1:** Patient demographics

	Total (*n*=45)	Maintained Immunity (*n*=34)	Lost Immunity (*n*=11)	*P*
Age at transplant, years	16.7 (12.7–18.5)	17.3 (13.4–19.0)	12.4 (3.9–17.4)	0.05
Sex				0.28
Male	29 (64.4)	20 (58.8)	9 (81.8)	
Female	16 (35.6)	14 (41.2)	2 (18.2)	
Race/Ethnicity				0.7
Hispanic	29 (64.4)	23 (67.6)	6 (54.5)	
Non-Hispanic White	9 (20.0)	6 (17.6)	3 (27.3)	
Non-Hispanic Black	5 (11.1)	4 (11.8)	1 (9.1)	
Asian	2 (4.4)	1 (2.9)	1 (9.1)	
Original disease				0.35
CAKUT	21 (46.7)	14 (41.2)	7 (63.6)	
FSGS	5 (11.1)	3 (8.8)	2 (18.2)	
Glomerulonephritis	10 (22.2)	9 (26.5)	1 (9.1)	
Other	9 (20.0)	8 (23.5)	1 (9.1)	
Deceased-donor renal transplant	40 (88.9)	31 (91.2)	9 (81.8)	0.58
Repeat transplant	2 (4.4)	1 (2.9)	1 (9.1)	0.43
Highly sensitized (PRA > 30%)	6 (13.3)	3 (8.8)	3 (27.3)	0.15
Induction agent				>0.99
Lymphocyte depleting agent	38 (84.4)	29 (85.3)	9 (81.8)	
Non-lymphocyte depleting agent	7 (15.6)	5 (14.7)	2 (18.2)	
Steroid-based immunosuppression	20 (44.4)	11 (32.4)	9 (81.8)	0.006
Follow-up time, months	14.9 (11.9–32.0)	22.8 (12.0–33.9)	12.6 (8.8–14.9)	0.07

*CAKUT* congenital anomalies of the kidney and urinary tract, *FSGS* focal segmental glomerulosclerosis, *PRA* panel of reactive antibodies

Values are expressed as n (%) or median (interquartile range)

Median time between confirmation of positive pre-transplant VZV IgG testing and transplant was 8.8 months (IQR 4.8–13.6), and there was no difference between the pre-transplant VZV IgG titers between the group who lost immunity vs. those who maintained their VZV IgG after transplantation, 1.7 index (IQR 1.3–3.4) vs. 1.9 index (IQR 1.6–2.6), respectively (Table 2, *P* = 0.99). Median time to negative post-transplant VZV IgG was 12.6 months (IQR 8.8–14.9) compared to the last VZV serological testing in those that maintained immunity was 22.8 months (IQR 12.0–33.9) (Table 2, *P* = 0.07). There were no differences in the WBC count at the time of the last post-transplant VZV IgG testing (Table 2, *P* = 0.92).

**Table 2:** Varicella zoster vaccination and IgG titer pre- and post-transplant

	Total (*n*=45)	Maintained Immunity (*n*=34)	Lost Immunity (*n*=11)	*P*
Number of pre-transplant VZV doses				0.35
1	2 (4.4)	2 (5.9)	0 (0)	
2	34 (75.6)	27 (79.4)	7 (63.6)	
3	7 (15.6)	4 (11.8)	3 (27.3)	
4	2 (4.4)	1 (2.9)	1 (9.1)	
Time between last VZV vaccine and transplant, months	98.1 (15.2–146.4)	103.9 (42.6–151.5)	10.7 (4.4–98.2)	0.02
Time between last VZV vaccine and transplant				0.01
≤ 12 months	10 (22.2)	4 (11.8)	6 (54.5)	
12–24 months	4 (8.9)	3 (8.8)	1 (9.1)	
> 24 months	31 (68.9)	27 (79.4)	4 (36.4)	
Time between pre-transplant VZV IgG and transplant, months	8.8 (4.8–13.6)	10.5 (5.1–13.7)	5.1 (3.3–15.3)	0.38
Pre-transplant VZV IgG, index	1.9 (1.3–2.57)	1.9 (1.6–2.6)	1.7 (1.3–3.4)	0.99
Time from transplant to post-transplant VZV IgG, months *	14.9 (11.9–32.0)	22.8 (12.0–33.9)	12.6 (8.8–14.9)	0.07
Post-transplant VZV IgG, index *	1.49 (1.1–2.3)	1.8 (1.3–2.5)	0.8 (0.6–1.0)	0.0001
WBC count, 1000/uL **	5.9 (4.6–7.4)	5.8 (4.6–7.2)	5.9 (3.0–7.4)	0.92

Values are expressed as n (%) or median (interquartile range). VZV, varicella zoster virus; IgG, immunoglobulin G; WBC, white blood cell

* The most recent VZV IgG or the first negative VZV IgG post-transplant

** WBC count at the time of post-transplant VZV IgG titer

### Induction agent used

There were no differences between the induction agents used among those who maintained vs. lost post-transplant VZV IgG immunity (Table 1). Nine of 11 subjects (81.8%) who developed negative VZV IgG post-transplant received a lymphocyte-depleting agent compared to 29 of 34 patients (85.3%) among those whose immunity persisted (*P* > 0.99).

### Number of VZV vaccines received prior to transplant

A majority of the patients received two doses of VZV vaccine prior to transplantation (75.6%); 2 subjects (4.4%) received only one dose of VZV vaccine, while 7 (15.6%) and 2 (4.4%) received three and four doses, respectively (Table 2). There is an increased risk for losing VZV seropositivity after transplant among those who required 3 or more doses of VZV vaccine in order to seroconvert prior to transplantation (HR 3.81, 95% CI 1.09–13.30, *P* = 0.04).

## Interval between last dose of VZV vaccine and transplant

**Fig. 1:**
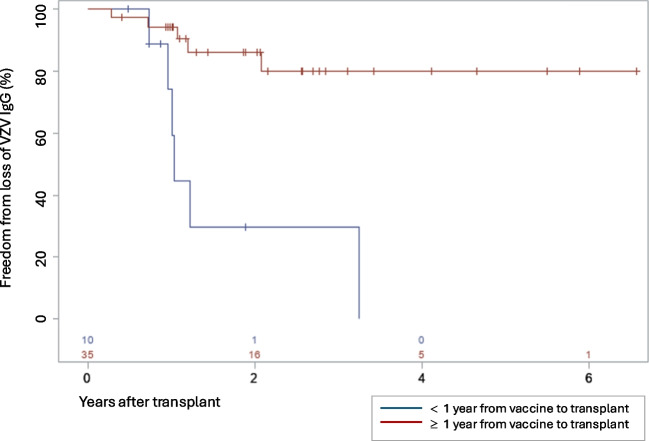
Kaplan–Meier curve for freedom from loss of VZV IgG among pediatric kidney transplant recipients who received their last VZV vaccine <1 year and ≥1 year prior to transplantation. The group differences were assessed by the log-rank test. *VZV IgG*, varicella immune globulin G

Time interval between last VZV vaccine dose and transplant was shorter among those who lost VZV immunity compared to those who maintained positive VZV IgG levels, 10.7 months (IQR 4.4–98.2) vs. 103.9 months (IQR 42.6–151.5) (Table 2, *P* = 0.02). Freedom from loss of VZV IgG up to 83 months following transplantation was compared based on the time interval between the last VZV vaccination and transplant (<  year vs. ≥1 year) (Fig. 1). Six of 10 (60%) of those who received their last VZV vaccine <1 year before transplantation lost their post-transplant VZV IgG seropositivity compared to 5 of 35 (14.3%) of those who were vaccinated ≥1 year prior (Table 2). Receiving VZV vaccination <1 year prior to kidney transplantation was associated with a higher risk for losing VZV immunity post-transplant (Fig. 1; HR 6.97, 95% CI 2.08–23.34). Furthermore, over a follow-up time of 3 years, 70.4% develop negative VZV IgG when vaccinated <1 year preceding transplantation compared to 20.0% in the cohort vaccinated ≥1 year earlier (Fig. 1, *P* = 0.0003).

### Post-transplant Varicella infection

Despite 24.4% of our post-transplant patients losing immunity to VZV, none of these patients developed VZV infection during the median follow-up period of 14.9 months (IQR 11.9–32.0 months).

## Discussion

Among our pediatric kidney transplant cohort with serologic response to VZV vaccination and no history of natural VZV infection before transplantation, 24.4% of recipients lost humoral immunity at a median time of 12.6 months post-transplant. Similar findings on the risk for loss of VZV IgG seropositivity are highlighted in several studies. Broyer et al. noted that among 94 pediatric patients who received VZV immunization prior to kidney transplantation, 7% and 24% had negative VZV IgG antibodies at 1- and 5-years post-transplant, respectively [12]. A meta-analysis on the seroprevalence of VZV IgG antibody in kidney transplant recipients illustrated varying VZV IgG durability post-transplant, ranging from 23 to 100% VZV IgG positivity between 657 and 2130 days following transplantation [14]. In a study of 18 pediatric solid organ transplant patients (8 liver, 6 kidney, 4 heart) with seropositivity of VZV IgG antibodies pre-transplant, 11% had undetectable VZV antibody titers within 6 months post-transplant [13]. Among pediatric liver transplant recipients, 42 of 67 (63%) who received the VZV vaccine prior to transplantation were non-immune to VZV at a median time of 3.3 years after transplantation [19].

We found no differences in the induction of immunosuppression received between those who maintained or lost VZV IgG following transplant. Patients who were maintained on steroid-based immunosuppression, however, were more likely to lose anti-varicella antibody at a median time of 13.0 months (IQR 10.3–20.1) post-transplant. In contrast, highly sensitized recipients (panel of reactive antibodies > 30%), all of whom received desensitization with a combination of plasmapheresis, IVIg, rituximab, and/or tocilizumab no more than 6 months prior to transplantation, did not appear to be at higher risk for losing VZV IgG antibody despite intensified immunosuppression going into transplant and maintained on steroid-based immunosuppression. It is important to note, however, that the highly sensitized patients do receive IVIg the day of and within a week following transplant, which could have provided additional VZV IgG. Although our cohort only had two patients who were treated for rejection before the loss of VZV IgG antibody, we could not statistically assess the correlation between treatment for rejection and the risk for loss of VZV IgG seropositivity. In a multicenter prospective study involving pediatric liver and kidney transplants, Barton et al. noted that while VZV IgG antibodies tended to be lower during periods of higher immunosuppression, the antibody levels remained protective [20]. Furthermore, one patient received a steroid pulse for treatment of acute rejection without subsequent decline in their VZV IgG titer [20].

While a majority (75.6%) of our cohort received 2 doses of VZV vaccine prior to transplantation, 4.4% received only 1 dose and 20.0% received 3 or more doses. Interestingly, those that received more doses than the standard childhood 2-dose VZV series were more likely to lose their VZV seropositivity following transplantation. Current recommendation from the Advisory Committee on Immunization Practices and Centers for Disease Control is a 2-dose VZV vaccine series due to the finding that a second dose of VZV vaccine produced a more robust antibody level compared to 1 dose, and decreased the rate of breakthrough varicella infection by 3.3-fold [23]. Nonetheless, primary vaccine failure after the 2-dose series is possible, necessitating an additional booster dose [24]. While the most likely cause of primary vaccine failure in our population are factors inherent to the immune response of the recipient, this could potentially also translate to a reason for the less durable VZV seroprotection in those requiring additional booster dose(s) [25].

The interval between the last dose of the VZV vaccine and transplantation appears to influence the durability of VZV IgG following transplant. Our cohort revealed that the threshold of <1 year best predicted a heightened risk for loss of anti-varicella antibody after transplantation. Similarly, in a multivariate analysis, Yoeli et al. also noted that a time interval of >1 year from the last vaccine to transplant was independently and significantly associated with the maintenance of immunity against VZV [19]. The authors hypothesized that the intensified immunosuppression immediately post-transplant could hinder the development of VZV immunity for those who had not yet mounted a full immune response following vaccination, potentially due to the short time interval between vaccination and transplantation [19]. Interestingly, a meta-analysis of adult transplant studies noted that some cohorts had 100% seropositivity even up to 1080 days post-transplant [14]. This raises the question of whether natural infection provides better VZV IgG durability compared to vaccination, as the adult cohorts are more likely to have had natural infection due to the lack of VZV vaccine during their childhood. It has been noted that vaccination typically results in lower levels of IgG antibody compared to natural infection [26]. In a sero-epidemiological study of varicella in an Italian cohort where newborn VZV vaccination has been mandatory since 2017, younger individuals had lower VZV IgG compared to older adults where VZV vaccination was not available (6–9 years 84.1% vs. 97.0% in >40 years) [27]. Nonetheless, virus-specific T cell proliferation contributes to one’s overall immunity, with evidence that both natural infection and vaccination induced CD4 + and CD8 + cytotoxic T lymphocytes at similar frequencies [28].

There are several limitations in our study, with one attributed to the small cohort of 45 patients, which raises the possibility for type 2 error. Post-transplant VZV IgG was checked at approximate defined timepoints, which may not accurately reflect the exact timing to loss of VZV IgG following transplantation. Given that our standard approach to assessing VZV IgG is with ELISA, we did not perform fluorescence antibody to membrane antigen (FAMA) testing, which has been identified to best correlate with susceptibility and protection against varicella disease [26]. As a result, we could have categorized those as having lost immunity when they still had sufficient protection against VZV, which could explain why we had no VZV disease in our cohort. It is important to note that immunocompetent children have also been shown to have declining VZV vaccine effectiveness over time. A case–control study of 339 children found that the overall effectiveness of the VZV vaccine was 97% in the first year after vaccination, which decreased to 84% two to eight years after vaccination [29]. Nonetheless, despite the decrease in the vaccine’s effectiveness in the second year after vaccination, breakthrough infections were mild among this cohort. Such decline in vaccine effectiveness can be associated with decreasing seroprevalence of VZV antibodies even among those who are not immunocompromised. In a study of 101 healthcare workers with documented 2 doses of varicella vaccine, 11.9% were found to be seronegative [30]. Furthermore, 11.5% of the seropositive healthcare workers produced low avidity antibody, reflecting suboptimal response to vaccination. In a case series of 182 healthcare workers with documented 2-dose varicella vaccine, 34% were seronegative for VZV IgG [24]. These data highlight the fact that vaccination-induced immunity can diminish over time among immunocompetent and immunosuppressed individuals and the proportion of those who lose VZV IgG are comparable between these two groups. As such, while none of our patients who lost their VZV immunity developed VZV infection despite being on immunosuppression, loss of VZV IgG may not lead to complete loss of VZV immunity, as viral-specific T-cell (Tc) responses could still be intact and provide some degree of immune protection [17, 31–34]. Future studies should evaluate VZV Tc and the clinical course in those who have and have not maintained post-transplant VZV IgG following pre-transplant VZV vaccination. Another limitation of this study stems from the unexamined risk factors that could influence our cohort’s findings, such as the course and duration of chronic kidney disease or dialysis, if patients received a preemptive transplant, and the use of pre-transplant immunosuppression.

## Conclusions

Pediatric kidney transplant recipients are at risk of losing VZV IgG seropositivity after transplantation. Post-transplant VZV serologies should be serially monitored to identify patients at high risk for disseminated VZV. Identifying these patients is important to counsel families on post-exposure prophylaxis or the need for immediate treatment should any symptoms arise. Given the risk of losing VZV IgG, close contacts should make sure they are up to date on vaccines to help provide herd immunity. Despite strategies for post-exposure prophylaxis and treatment of VZV infection in solid organ transplant recipients, it does not offer complete protection against the morbidity and mortality associated with VZV disease in the immunocompromised. Further research should be directed at evaluating VZV cellular immunity and determining strategies to provide protection against VZV among pediatric kidney transplant recipients who lose their pre-transplant vaccine-induced humoral immunity.

## Supplementary Information

Below is the link to the electronic supplementary material.

ESM 1 Graphical Abstract (PPTX 165 KB)

## Data Availability

The datasets generated during and/or analyzed during the current study are available from the corresponding author on reasonable request.

## Abbreviations

ATG: Anti-thymocyte globulin
IgG: Immune globulin G
LDA: Lymphocyte depleting agent
NLDA: Non-lymphocyte depleting agent
Tc: T-cell
VZV: Varicella zoster
WBC: White blood cell
