# Supplementary material for: Risk factors for loss of Varicella immunity after pediatric kidney transplantation
Source: Pediatr Nephrol. 2025 Nov 13;41(3):837–44. doi: 10.1007/s00467-025-07022-7 (PMC12852294; doi:10.1007/s00467-025-07022-7)
Supplement: Supplementary file 1 — Graphical Abstract (PPTX 165 KB) [file 467_2025_7022_MOESM1_ESM.pptx]

## Slide 1
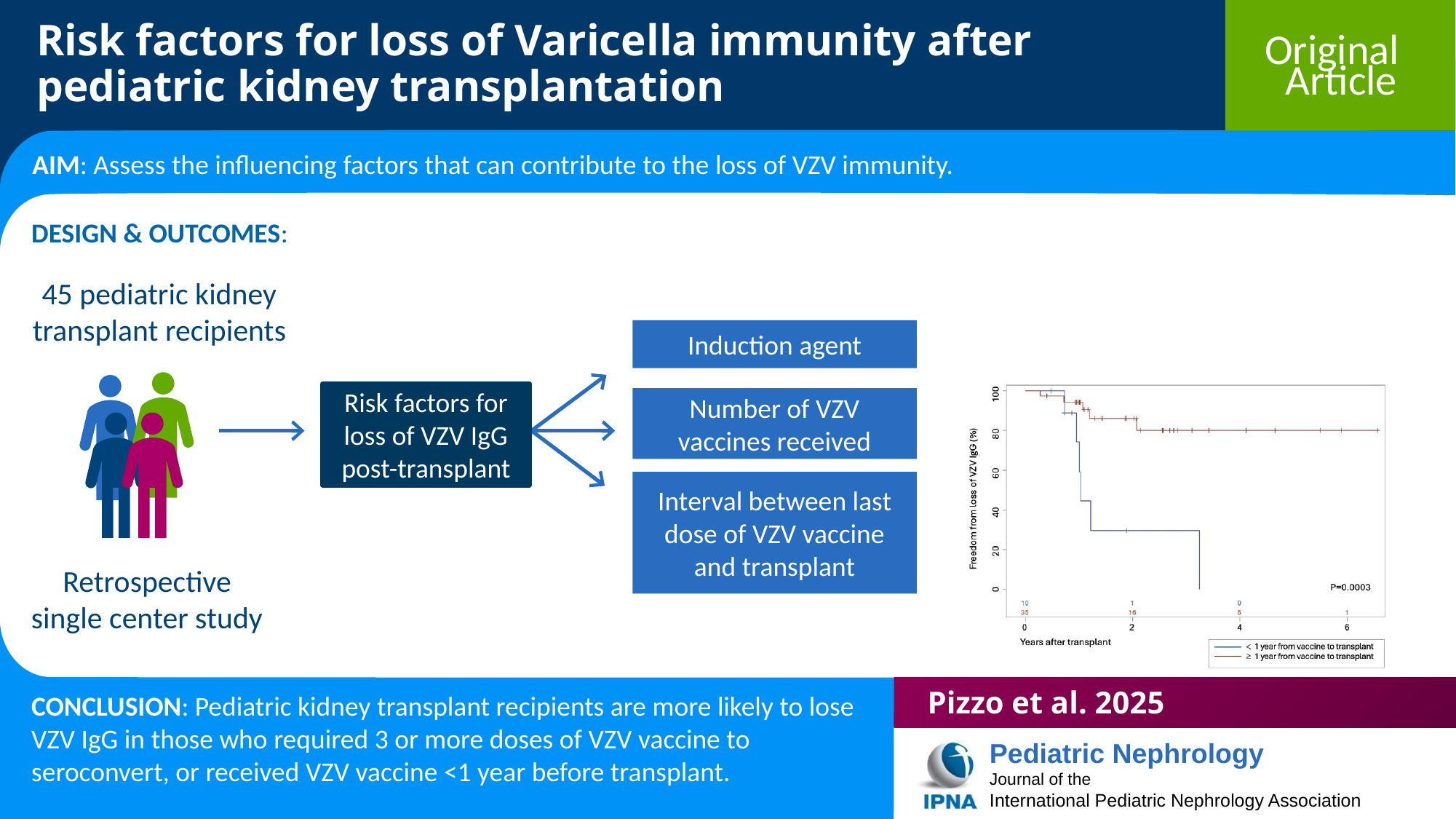

Risk factors for loss of Varicella immunity after pediatric kidney transplantation
AIM: Assess the influencing factors that can contribute to the loss of VZV immunity.
DESIGN & OUTCOMES:
45 pediatric kidney transplant recipients
Induction agent
Risk factors for loss of VZV IgG post-transplant
Number of VZV vaccines received
Interval between last dose of VZV vaccine and transplant
Retrospective single center study
Pizzo et al. 2025
CONCLUSION: Pediatric kidney transplant recipients are more likely to lose VZV IgG in those who required 3 or more doses of VZV vaccine to seroconvert, or received VZV vaccine <1 year before transplant.
